# Novel method of building train and test sets for evaluation of machine learning models related to software bugs assignment

**DOI:** 10.1038/s41598-023-48617-0

**Published:** 2023-12-06

**Authors:** Lukasz Chmielowski, Michal Kucharzak, Robert Burduk

**Affiliations:** 1 Nokia Solutions and Networks sp. z o.o., 02-685 Warsaw, Poland; 2 Wroclaw University of Science and Technology, 50-370 Wroclaw, Poland

**Keywords:** Computer science, Software

## Abstract

Nowadays many tools are in use in processes related to handling bug reports, feature requests, supporting questions or similar related issues which should be handled during software development or maintenance. Part of them use machine learning techniques. In introduction is presented a review of fundamental methods used for evaluation of machine learning models. This paper points out weak points of currently used metrics for evaluation in specific context of the cases related to software development especially bug reports. The disadvantages of state of the art are related to disregarding time dependencies which are important to be applied for creating train and test sets as they may have impact on results. Extensive research of the art has been conducted and has not been found any article with the use of time dependencies for evaluation of machine learning models in the context of works related to software development applications like machine learning solutions to supporting bug tracking systems. This paper introduces a novel solution which is devoid of these drawbacks. Experimental research showed the effectiveness of the introduced method and significantly different results obtained compared to the state-of-the-art methods.

## Introduction

### Background of the study

During the development of various types of systems, including software and those related to the hardware part, it is inevitable to make mistakes. In the event of noticing unexpected behavior of the system, testers or users create bug reports. Such a report may contain the contents of the log, screenshots, photos, reports from the spectrum analyzer, etc. Reporters should include information related to the discrepancy between the expected operation of the solution and the actual results obtained. This discrepancy may be the result of, e.g., a software malfunction, hardware failure, or environmental factors. Such a report must be assigned to a group of engineers for further analysis. This activity can be supported by machine learning solutions.

### Problem statement

The paper discusses different methods of evaluation of results of machine learning predictions related to reports of bugs, feature requests, supporting questions or similar related issues which should be handled during software development or maintenance. For instance, it may be evaluation of machine learning predictions of bug reports assignments. There is plethora of ways to classify issue or bug report as for instance classify severity in article^1^ or assign it to group in which should handle cases in papers^2,3^. The problem raised in the article concerns about use for evaluation in these specific applications time dependencies with usage of for instance: date of issue creation, date of solving issue or assigned states with corresponding dates of changes. Time from creation bug report to solving the case may, contrary to appearance, take a long time. For example, it may take a day or two to resolve a problem, but on the other hand, some cases are resolved after more than a year. Transition states might be also used in cases like an issue that has been marked as solved and later reverted from that state due to finding that delivered fix had been working only partially.

### Organization of the manuscript

The paper is organized as follows. Section Introduction contains information about background of the study, problem statement, related works, motivation including research gap. At the end of this part, the work contribution and its significance are shortly summarized. Next section, Methods, begins with ways of presenting machine learning results with the use of confusion matrix and description of state-of-the-art methods for building train and test sets in the context of software bug reports assignment. The section also discusses novelty in building train and test sets in the context of software bug reports assignment. The paper ends with sections Results and Discussion and Conclusion.

### Related works

There are plenty of publications related to handling of reports of bugs, feature requests, supporting questions or similar related issues which should be handled during software development or maintenance. None of those publications consider the influence of time dependencies related to date of reporting and solving software bug reports on evaluation methods which are used in them. However, in these publications, state-of-the-art methods that are not suitable for evaluation of machine learning models related to software bug reports have been used to evaluate machine learning tasks. Currently different approaches are being used, for instance precision used in this work is about a bug mining tool to identify and analyze security bugs using Naive Bayes and TF-IDF^4^. Combination of metrics like accuracy, precision and recall are applied in analyses with the aim to detect bug report duplication^5^. As there were no details about ways of data splitting into train and test sets, there were probably applied default assumption about random split. More advanced train and test sets creation methods, e.g., cross-validation, are also applied in problems related to software bug report assignment. As another example, there is a need to predict whether the first assignment of bug report is likely to be reassigned in the future^2^. In the second example^6^, Latent Semantic Indexing for reduction of the dimensionality and Support Vector Machine for triaging bug reports is applied. Time based activity profiling of developers for creation of time oriented expertise model were investigated in paper^3^ where top-k accuracy metric was used. Metric which takes into consideration a couple of best predictions was also used in work^7^ which utilizes data from two types of inputs. Natural language description and discrete features separately. On non-textual inputs Principal Component Analysis is applied. For text data is utilized Entrophy-based feature selection. Bidirectional Long Short-Term Memory Network was considered to automate the bug triaging process in publication^8^. In material^9^ standard methods for evaluation on train, test set are applied, but separated based on time dependencies in that way that reported earlier were used for train set and reported later used for test set. The problem of bug report duplicates is mentioned in works^10,11^. The first is about possibilities of reducing redundant bug records, whereas the second about risk estimation among others considers predicting bug fix time. Publication^12^ presents the statement that even after application of solution for just-in-time retrieval solution to avoid duplicates being created, there is still over 10% of bug duplicates in described Mozilla Projects. Authors of paper^13^ even divides duplicated bug reports into two different categories. The first is related to reported bugs duplicates the master report of the same issue while the master problem was not yet resolved. The second category when during the report of issue master issue was already solved. Other works in the field of technical information technology and telecommunications where time dependencies are important are articles related to Quality of Service where topics like and latency re-transmision of data packets^14^, latency^15^ are considered.

### Motivation and research gap

The research gap is strictly related to impact of time dependencies related to software bug creation and resolution dates with the use of machine learning techniques. Current methodologies do not employ these time dependencies. They are significant due to the fact that, in general, the problems solved in each department are expected to be similar to some extent, but we must bear in mind that the characteristics of the reported faults by software users change over time. The problem related to time dependencies is considered due to the fact that during software development, its behavior, the flaws it possesses, or its characteristics change. For instance, introduction into developed application new functionalities which are expected by the customers is accomplished by modification of existing source code. Therefore, in such cases new error numbers, configuration parameters, patterns of messages, alerts etc. may be introduced. Those parts when creating machine learning models lead to creating new features in representation of data like feature vector shown in Eq. (1). For instance, related to new types of configuration parameters may be then introduced as new terms in term frequency representation. In real use applications from creating bug reports to solving ones take time. The models for production use are trained only with the use of resolved cases. Therefore, data representations used for creation of model for predictions at the beginning of introduction of each new functionality will miss at the time of creation of the first bug reports specific features related to them in vector representation like in Eq. (1).

1 $$\begin{aligned} X = [x_{1} \; x_{2} \; x_{3} \; \ldots \; x_{N}] \end{aligned}$$

The new features will not be introduced into representation until the first case is resolved and used for training of model for production purposes. Before that time, some similar cases may be reported, in real case application all of these will be predicted with the use of model being trained without those described examples on data which were currently possessed and labelled. Common approaches in ML applications utilize randomized ways of creating train and test sets. It may lead to a situation in which different samples referring to the same of similar case which were reported nearby will be present in both train and test set what is not possible in real case applications due to the above-mentioned restrictions. Therefore, the results of evaluation where those restrictions are disregarded may be significantly different. Those approaches should not be used for evaluation in those applications if the aim is to get results similar to that which we can obtain in production of solution. The main advance of the proposed method in paper being verified is that it reflects possible real-world scenarios.

Two research questions were explored:

- Are the standard machine learning methods for evaluation appropriate to evaluate problems related to bug handling?
- If not then what experimental protocol should be introduced?

Hypothesis stated:

*The state-of-the-art methods of building train and test sets may be not appropriate for evaluation of problems related to software bug reports assignment.*

### Main contributions of research

The paper shows that state-of-the-art methods are not appropriate for evaluation of machine learning models in the context of cases related to software bug reports. Current solutions disregard time dependencies like creation and resolving date of issues related to software bug reports. What is not appropriate especially in case of results of predictions of software bug report assignment if the aim is to estimate what kind of results are possible to obtain in real production use. The outcomes of work are results of scientific research related to introduced in this paper original and innovative solutions of the scientific problem of evaluation of machine learning models in the context of software bug reports assignment. Introduced in this paper methods related to including time dependencies into evaluation of machine learning models in the context of software bug reports assignment do not impact accuracy of production solutions itself, however thanks to them the results better reflects real use cases. The presented innovative original solution in the field of application of research results are significant for the economic sphere.

## Methods

### Presenting machine learning results with the use of confusion matrix.

Confusion matrix is used for presenting information related to results of machine learning predictions. In columns are presented predicted classes, in rows actual classes. That way of orientation of matrix is used in many sources^19–26^, however different sources^27–29^ use another. This means that adding the right headings in this kind of presentation is very important to avoid misunderstanding. Additionally normalized way of confusion matrix is presented in Table 1. By normalization means the division of each element in the matrix by the sum of the samples $\left( \sum {X} \right)$.

**Table 1:** Normalized confusion matrix for multiclass problems.

	Predicted class			
	Department K	Department L	Department M	Department N
Actual class				
Department K	$\frac{X_{KK}}{\sum {X}}$	$\frac{X_{KL}}{\sum {X}}$	$\frac{X_{KM}}{\sum {X}}$	$\frac{X_{KN}}{\sum {X}}$
Department L	$\frac{X_{LK}}{\sum {X}}$	$\frac{X_{LL}}{\sum {X}}$	$\frac{X_{LM}}{\sum {X}}$	$\frac{X_{LN}}{\sum {X}}$
Department M	$\frac{X_{MK}}{\sum {X}}$	$\frac{X_{ML}}{\sum {X}}$	$\frac{X_{MM}}{\sum {X}}$	$\frac{X_{MN}}{\sum {X}}$
Department N	$\frac{X_{NK}}{\sum {X}}$	$\frac{X_{NL}}{\sum {X}}$	$\frac{X_{NM}}{\sum {X}}$	$\frac{X_{NN}}{\sum {X}}$

### State-of-the-art methods for building train and test sets

In case of classification problems in machine learning to train model is used a set of data called train set. To test model is used a set of data called test set. Especially for applications related to neural networks sometimes is also used third type of set called validation set. The purpose of using that set may be for instance to check the state of neural network every epoch during the training phase and decide whether the training of the network meets condition for early stopping. In that situation the state of network is saved and used for further evaluation on separate test set, which has never been used during the test or evaluation phase of model.

#### Standard train test splitting

During common creation of train and test set for evaluation usually dataset is split into train and test set randomly.

#### Stratified train test splitting

The stratified version for division into multiple sets uses the information about classes and tries to keep the radio between the classes in sets as much as possible like each other.

#### No shuffle

In implementations like in library Scikit-learn^16^, there is an option to use a split of the dataset for train and test without shuffling. That might be applied in an application where the order of samples matters.

#### k-Fold Cross-Validation

**Figure 1:**
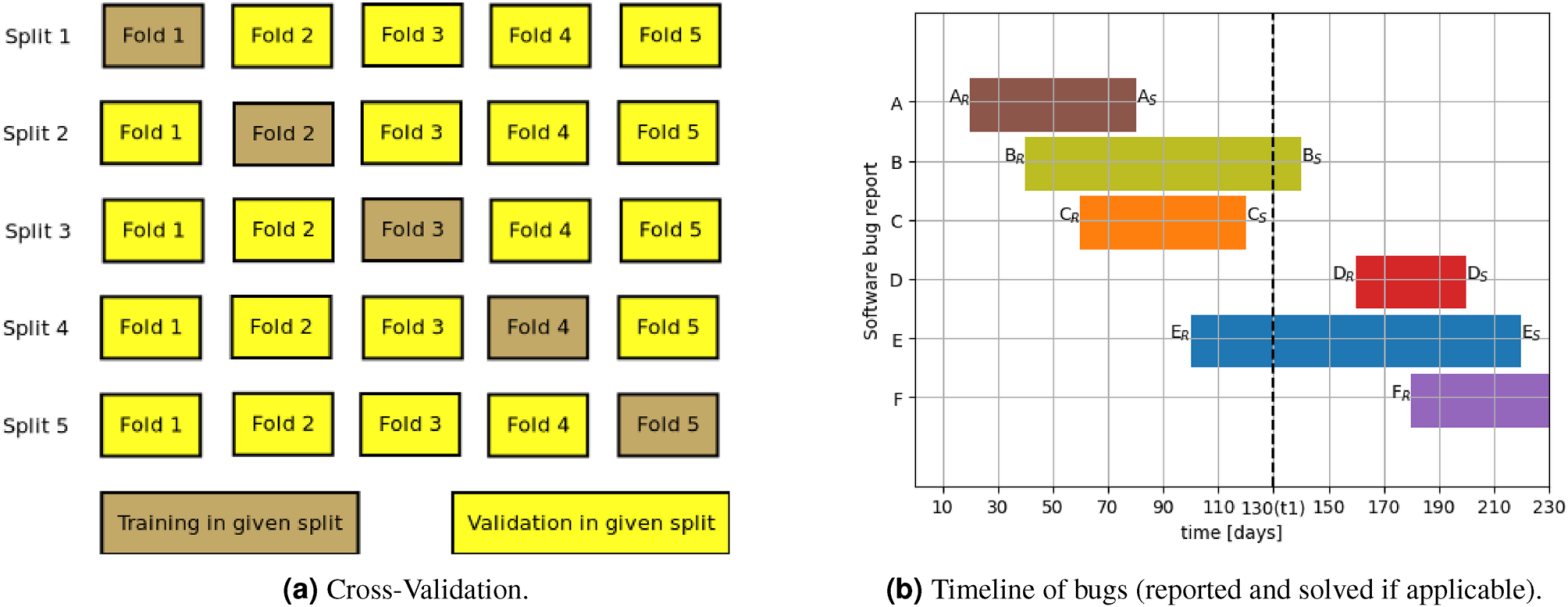
Cross-Validation and timeline of bugs (reported and solved if applicable).

Cross-validation is a procedure used to evaluate machine learning models which uses many splits on data. Generally, data is divided into k sets usually called folds. For each split one set is chosen as validation set, and the rest of data is used for training model in given split. After getting results for each split the results are summarized. Example of visualization of splits is shown in Fig. 1a^17^.

#### Leave one out cross-validation

This procedure might be useful especially for small datasets. In that case the number of folds equals the number of samples in the dataset. The model is tested on single sample while trained using the rest of available data^18^.

### Novelty in building train and test sets in the context of software bug reports assignment

**Figure 2:**
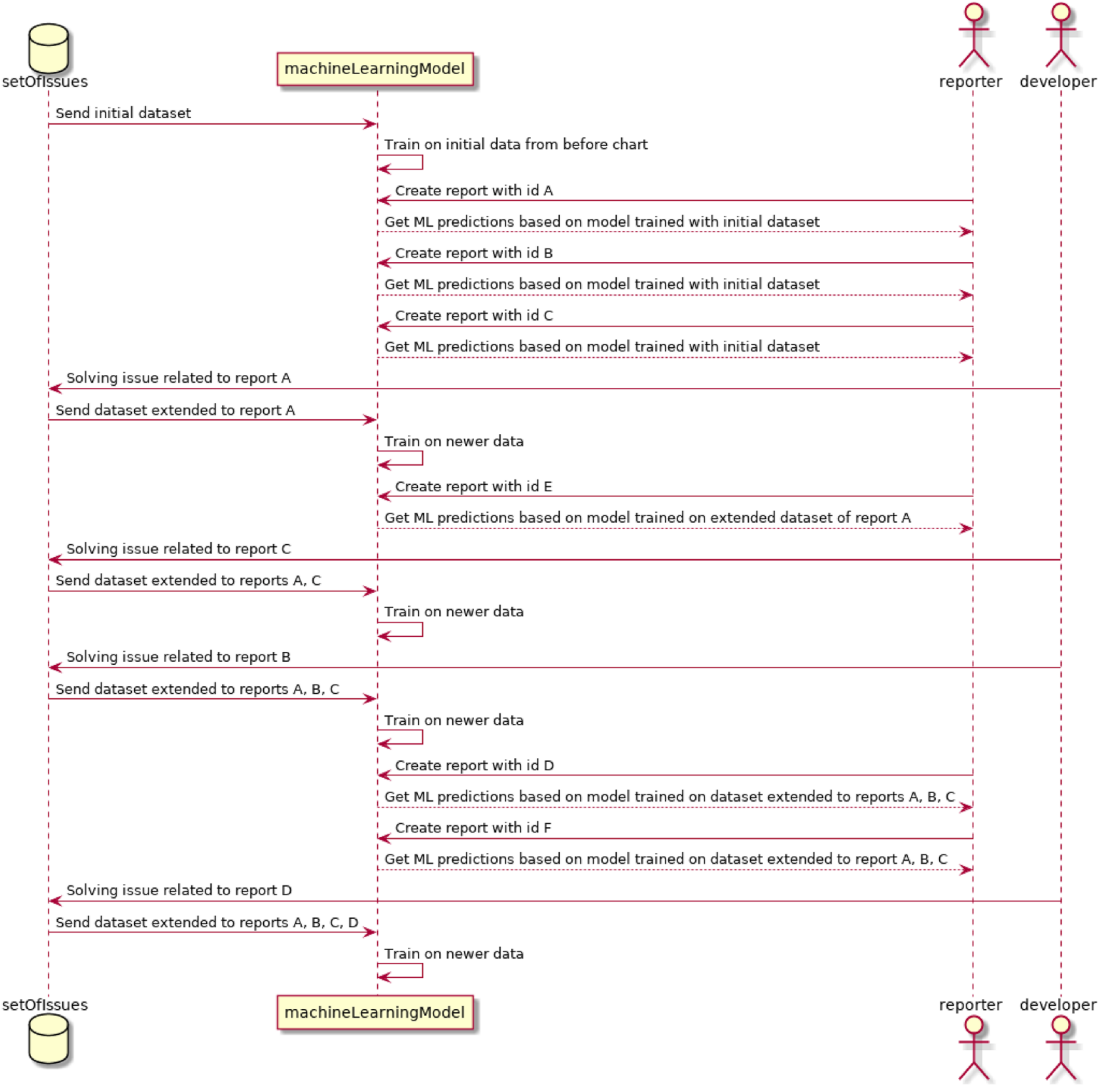
Sequence diagram presenting time dependencies of real use case in the context of solution related to software bugs assignment systems.

The novelty is about specific splitting of train and test sets for purpose of evaluation to be more adequate and like results which we could expect from working production setup. While preparing model for production mode usually only solved cases with respective final correct labels are used for creating train set. The advance is taking for evaluation cases separated with the use of time dependencies. An example of data dependency is shown in Fig. 1b. For sake of simplicity of presentation each case is identified with the id like *A*, *B*, *C*, ...It is only a identifier and it is not a label/class in the context of Machine Learning task. Figure 1b shows an example diagram with issues named *A*, *B*, *C*, *D*, *E*, where $A_{R}$ is point where case *A* was reported, $A_{S}$ is a point where case A was solved. The proper label/class related to group(s) which were engaged in solving case used for training can be assigned after the case is solved. According to this example the split point is marked as $t{_1}$. For train data cases *A*, *C* were selected as were solved before $t_{1}$ and for test set selected cases reported after $t_{1}$ (*D*, *F*). However, in some applications software bug reports which were reported before $t_{1}$, but not solved at the time of prediction might utilize machine learning supporting solutions to point out the proper group. Sequence diagram has been presented in Fig. 2. There are showed interactions in chronological order between objects called setOfIssues, machineLearningModel, reporter, and developer. Diagram clearly shows accessible data over time which can be utilized for model training. Dataset of issues is updated there by developer at the time of resolving issues when the labels are assigned, what triggers retraining of model which can be accessed by reporter. More complex solutions may utilize also the time needed for real use application to deliver new model for production $\Delta t$. For evaluation it might be also used with derived approaches using results from multiple splits by moving time division point or moving windows using this fact. After calculating results for multiple splits, they may be averaged or analyzed in another way for better presentation of results. These time dependencies are important for evaluation especially when taken into consideration duplicates and duration of solving issue related to bug report. In case of random split and other standard machine learning methods for creating train and test sets like these in source^16^, these time dependencies are skipped. This fact may impact on results, because model used for production purpose cannot be trained with the recently reported or not yet resolved bug reports that situation is not the case in random split evaluation especially when there is a lot of bug report duplicates. We should expect that in general when applying these time dependencies, the results which would be obtained will be worse than with standard methods which do not meet those requirements like Cross-Validation. At the same time, we have to remember that the results which were obtained using methods which do not meet the requirements of time dependencies do not show results which are similar to that what might be obtained in production of such solutions.

In practical applications such models may be retrained daily or even more frequent to minimize the effect. Moreover, in such cases the window which is used for training might be fixed by the beginning date, time duration, number of samples in the window or even more complex to somehow adjust the distribution of classes inside training set.

#### Set of novel methods

2 $$\begin{aligned}{} & {} \mathop {S}\limits ^{t_{S}}_{t_{R}} \;; \; t_{S} - \mathrm {date \, of \, solving} \;; \; t_{R} - \mathrm { date \, of \, reporting } \;; \; S - \mathrm { Software \, Bug \, Report } \end{aligned}$$

3 $$\begin{aligned}{} & {} \mathbb {X} = \left\{ \mathop {S}\limits ^{t_{S}}_{t_{R}}: t_{S} < t_{D}\right\} \;; \; \mathbb {X} - \mathrm {Train \, Set} \;; \; t_{D} - \mathrm {time \, of \, division} \end{aligned}$$

4 $$\begin{aligned}{} & {} \mathbb {Y} = \left\{ \mathop {S}\limits ^{t_{S}}_{t_{R}}: t_{R} > t_{D} \right\} \;; \; \mathbb {Y} - \mathrm {Test \, Set} \end{aligned}$$

5 $$\begin{aligned}{} & {} \mathbb {Z} = \left\{ \mathop {S}\limits ^{t_{S}}_{t_{R}}: t_{R} < t_{D} \wedge t_{S} > t_{D}\right\} \;; \; \mathbb {Z} - \mathrm { Spare \, Data } \end{aligned}$$

##### Single division point

In the novel approach metric for evaluation of machine learning models related to software bug reports assignment is being calculated with the use of single split point $t_{D}$ used for building train and test sets for evaluation. Introduced symbols and general definitions of sets are presented in Eqs. (2) to (5). Division point $t_{D}$ can be selected arbitrarily, e.g. $t_{D}={t_{p80}}$, where $t_{p80}$ is a date of reporting case in about 80th percentile of dates of creation. Let assume that the calculation of metric in single point will be symbolized with $\lambda _{metric}\left( t_{D} \right)$ for instance $\lambda _{acc}\left( t_{p80} \right)$ (See Eq. 6). The point $t_{1}$ does not have to be chosen randomly, it may be selected in a different ways depending on needs.

6 $$\begin{aligned} acc = \lambda _{acc}\left( t_{p80} \right) \end{aligned}$$

##### Multiple division points

There might be multiple solutions for averaging metric with the use of multiple division points, for every point or for instance with the use of moving window which can be defined both by time, or number of issues, or even some kind of stratification. In Eqs. (7) to (10) is defined example with the use of division for every reasonable point. However, in practice, the starting point should not be one of the first, given the chronological order.

7 $$\begin{aligned}{} & {} \mathbb {T} = \left\{ t_{R}: \underset{S \in \left\{ \mathbb {X}, \mathbb {Y} \right\} }{\forall } \mathop {S}\limits ^{t_{S}}_{t_{R}} \right\} \cup \left\{ t_{S}: \underset{S \in \left\{ \mathbb {X}, \mathbb {Y} \right\} }{\forall } \mathop {S}\limits ^{t_{S}}_{t_{R}} \right\} \end{aligned}$$

8 $$\begin{aligned}{} & {} \mathbb {Q} = \mathbb {T} \setminus \{min\left( \mathbb {T} \right) , max\left( \mathbb {T} \right) \} \end{aligned}$$

9 $$\begin{aligned}{} & {} metric = \frac{\sum _{t\in \mathbb {Q}} \lambda _{metric}\left( t \right) }{card\left( \mathbb {Q}\right) } \end{aligned}$$

10 $$\begin{aligned}{} & {} acc = \frac{\sum _{t\in \mathbb {Q}} \lambda _{acc} \left( t \right) }{card\left( \mathbb {Q}\right) } \end{aligned}$$

### Description and experimental protocol used to evaluate novelty

For below described experiments were performed calculations with four different methods of evaluation:

- split for train and test set randomly with shuffle of data (20% for test data);
- Cross-validation (5 folds);
- split for train and test set with the use of only date of reporting (8 months for train set, following 2 months for test set);
- split for train and test set with the use of novelty so both data of reporting and solving was taken into consideration (8 months for train, following 2 months for test).

Each experiment contains data from the range of 10 months. Please note that in the last of evaluation methods cases reported within the first 8 months and resolved later cannot be taken into consideration and were removed. The task performed during the experiments is to assign the report of bug to proper department responsible for investigation or solving issue. For performing that research only cases where fixes have been delivered have been taken into consideration. All calculations have been performed with the same way of preprocessing, with the same parameters to build TF-IDF representation. As a finial algorithm to assign department was used Logistic Regression. For each setting described above 10 series of calculations were performed with the move of dataset by one month between series.

## Results and discussion

**Figure 3:**
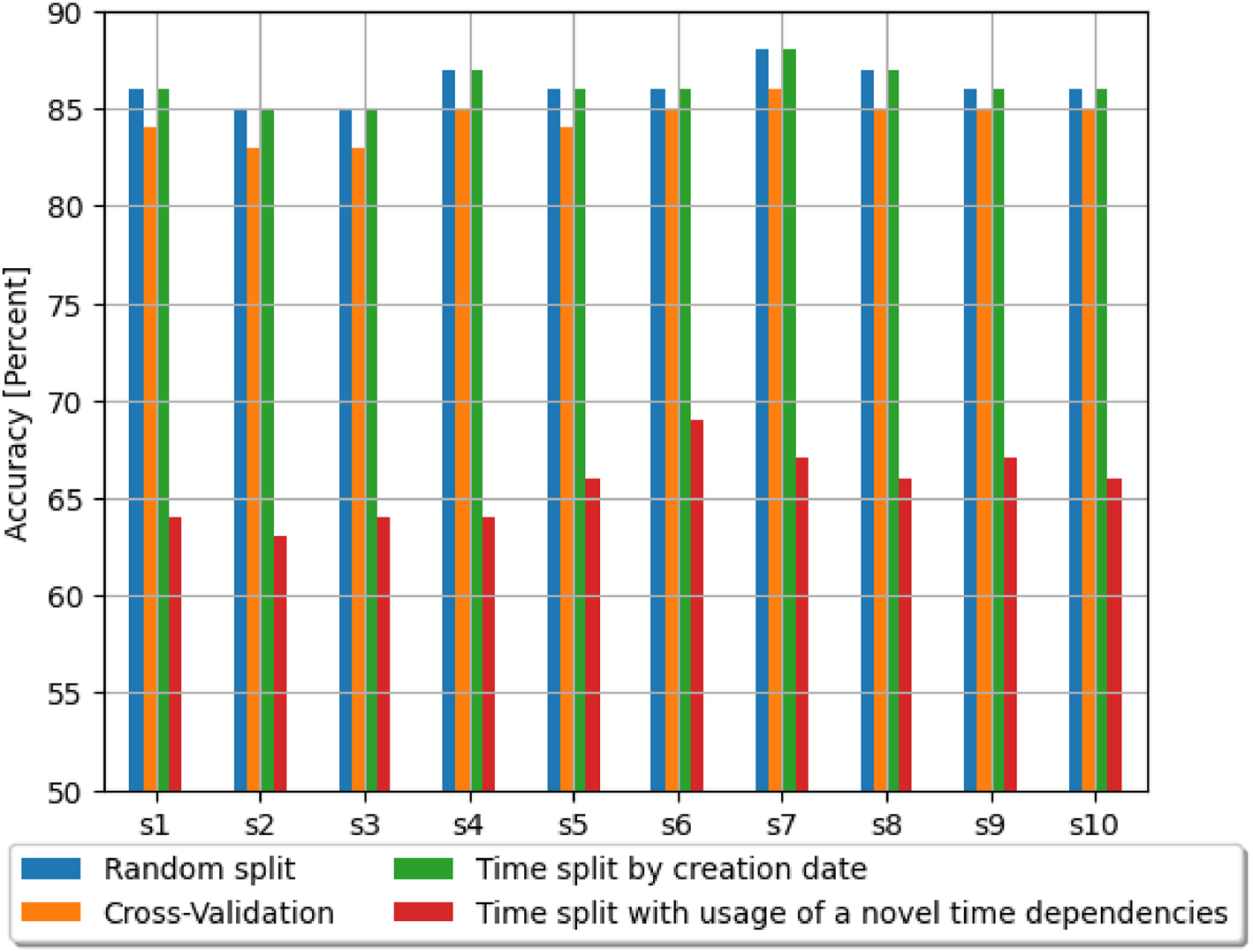
Comparison of accuracy.

**Figure 4:**
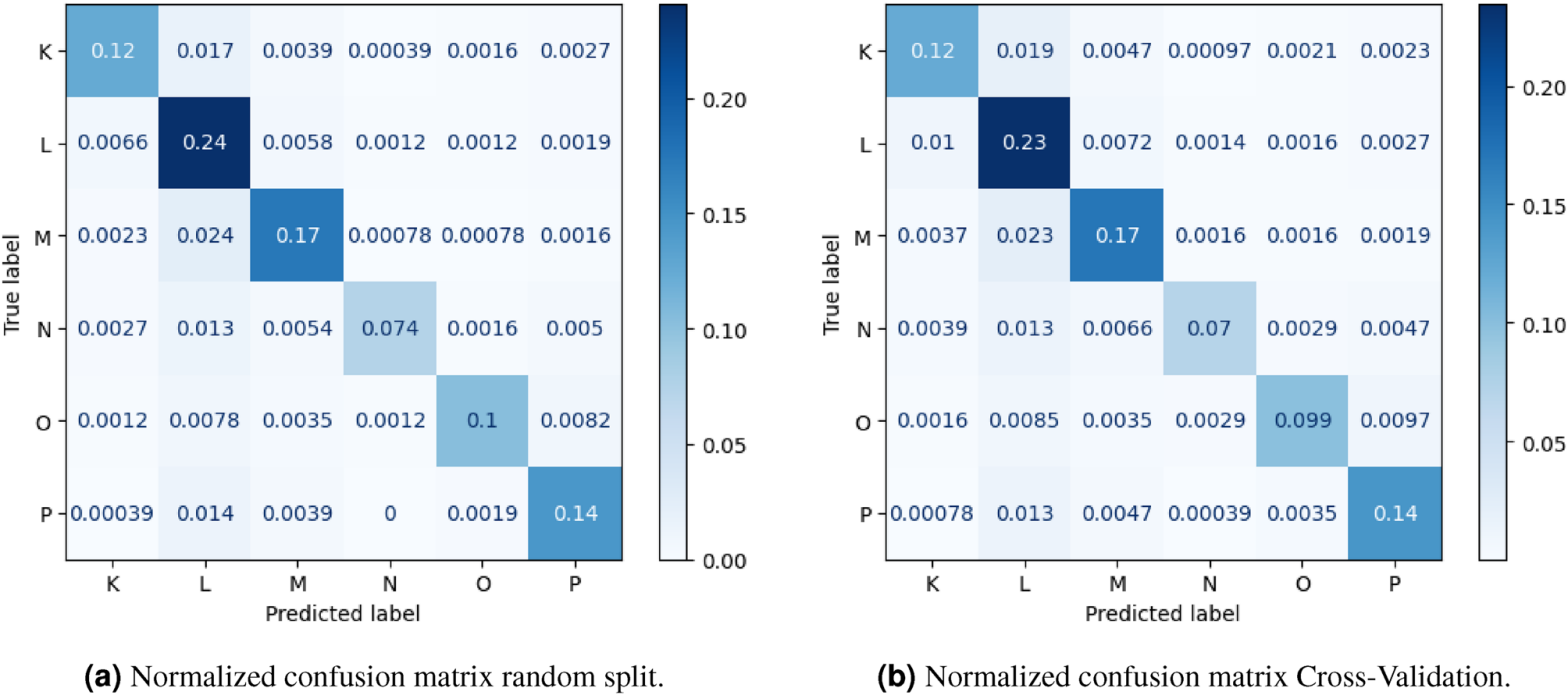
Normalized confusion matrix random split, and normalized confusion matrix Cross-Validation.

**Figure 5:**
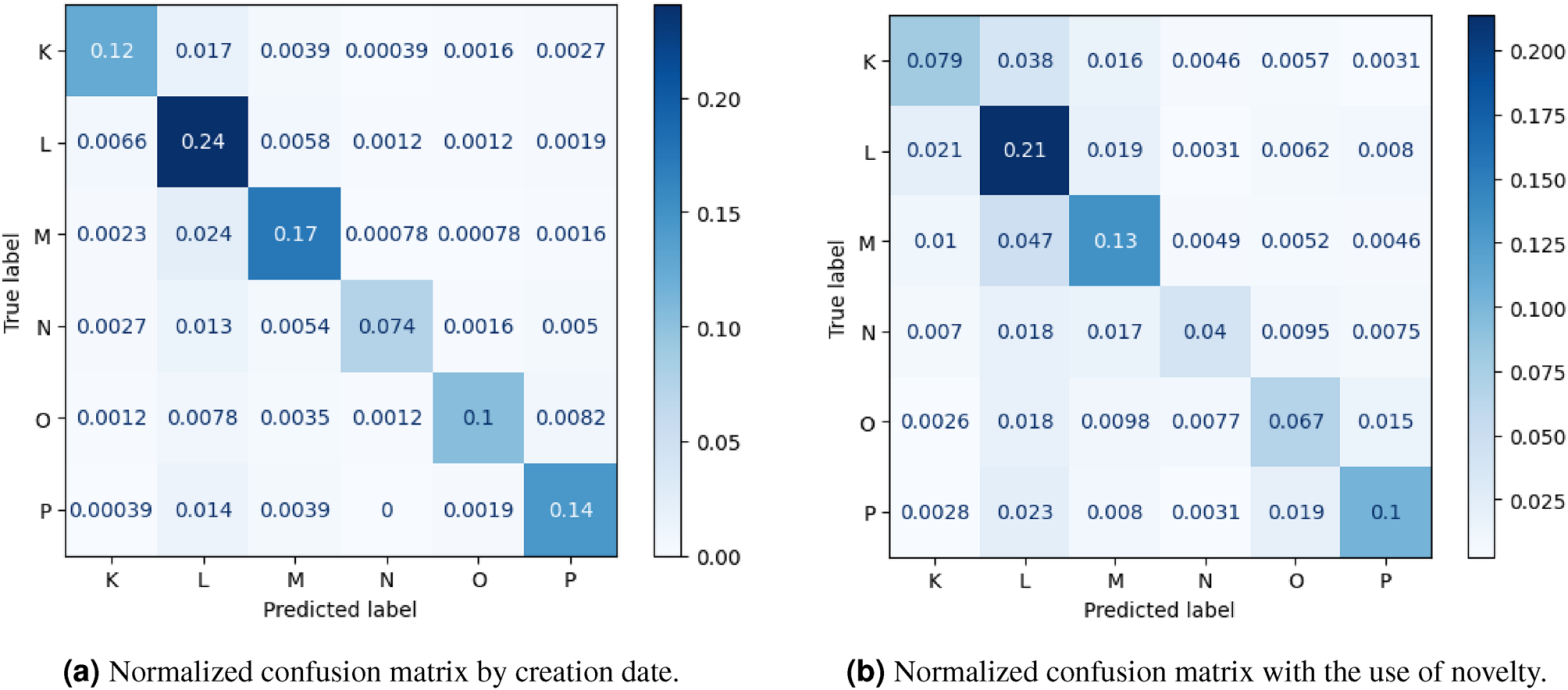
Normalized confusion matrix by creation date, and normalized confusion matrix with the use of novelty.

Table 3 contains the results with the random split with shuffling. The measures which were presented are accuracy, weighted precision and weighted recall. The weight is related to the number of samples. Table 4 presents accuracy in case of Cross-Validation. Accuracy in the case of splitting data for train and test set with the use of time dependencies is shown in Tables 5 and 6. First of them with the only use of date of reporting of report, second with use of novelty for date of solving. Although the results of evaluation based on time split by creation dates includes dependencies relating to date of creation, they disregard the time of resolving the issues, therefore they do not obey the laws of physics. For each of ways of evaluation for the first series results are presented in normalized confusion matrices (Figs. 4a,4b, 5a,5b). From the results we can clearly notice that results with the use of novelty are significantly different than the rest of results which have been obtained. Comparison of accuracy has been also shown in the chart (Fig. 3) and Table 2 for sake of transparency. For all series the results gathered with the use of novel method includes time dependencies between dates of creation and resolution of software bug report prediction accuracy is lower by at least fifteen percentage points by methods disregarding them. That novel method of building train and test sets for evaluation of machine learning models is the only one from those taken to comparison which meets the real use conditions. Mentioned dependencies are related to dates of creation and resolving the case. The model for evaluation should be trained only with cases which have labels assigned (in that case were resolved), before date of possible real usage in production (in that case date of creation software bug report). Noticing this fact and knowing that this method better reflects the production conditions of the applications of these methods, the thesis is put forward that it is better to use the method related to time dependencies and introduced novelty, if the aim is to reflect the results that can be achieved in real use.

**Table 2:** Comparison of accuracy.

Series	Random split	Cross-Validation	Time split by creation date	Time split with the usage of a novel time dependencies
s1	0.86	0.84	0.86	0.64
s2	0.85	0.83	0.85	0.63
s3	0.85	0.83	0.85	0.64
s4	0.87	0.85	0.87	0.64
s5	0.86	0.84	0.86	0.66
s6	0.86	0.85	0.86	0.69
s7	0.88	0.86	0.88	0.67
s8	0.87	0.85	0.87	0.66
s9	0.86	0.85	0.86	0.67
s10	0.86	0.85	0.86	0.66

**Table 3:** Detailed results of random split.

No.	Accuracy	Precision	Recall
1	0.86	0.87	0.86
2	0.85	0.86	0.85
3	0.85	0.86	0.85
4	0.87	0.88	0.87
5	0.86	0.87	0.86
6	0.86	0.87	0.86
7	0.88	0.89	0.88
8	0.87	0.87	0.87
9	0.86	0.87	0.86
10	0.86	0.88	0.86

**Table 4:** Detailed results of Cross-Validation.

No.	Accuracy	Precision	Recall
1	0.84	0.84	0.84
2	0.83	0.84	0.83
3	0.83	0.84	0.83
4	0.85	0.85	0.85
5	0.84	0.84	0.84
6	0.85	0.85	0.85
7	0.86	0.87	0.86
8	0.85	0.85	0.85
9	0.85	0.86	0.88
10	0.85	0.86	0.85

**Table 5:** Detailed results of split with use of the novelty.

No.	Accuracy	Precision	Recall
1	0.86	0.87	0.86
2	0.85	0.86	0.85
3	0.85	0.86	0.85
4	0.87	0.88	0.87
5	0.86	0.87	0.86
6	0.86	0.87	0.86
7	0.88	0.89	0.88
8	0.87	0.87	0.87
9	0.86	0.87	0.86
10	0.86	0.88	0.86

**Table 6:** Detailed results of split with use of the novelty.

No.	Accuracy	Precision	Recall
1	0.64	0.64	0.64
2	0.63	0.64	0.63
3	0.64	0.65	0.64
4	0.64	0.65	0.64
5	0.66	0.67	0.66
6	0.69	0.69	0.69
7	0.67	0.68	0.67
8	0.66	0.66	0.66
9	0.67	0.68	0.67
10	0.66	0.67	0.66

## Conclusion

The paper summarizes different methods of evaluation of machine learning models in the context of problems related to software bugs. Commonly used machine learning evaluation methods like random split, Cross-Validation and even standard splitting based on time like for instance based on date of creation of problem reports does not include the time of solving issue what may have serious impact on results. In the paper was introduced a proposition to create train and test sets built based on time dependencies to create test set with bug report created not earlier than the latest date of solving of bug report from train set. The main advantage is that the results come from predictions in simulations which better reflect real use. Please note that although the results with the use of novelty may be significantly worse as they are in that case, the other ones are not reasonable due to breaking time requirements and should not been applied for such cases. Experimental results which were conducted in that work clearly show the difference between evaluation with the use of novelty and standard methods for general classification problems. Authors claim that the rest of the methods which do not meet time dependencies are not appropriate for evaluation problems related to software bug reports as they do not respect real time dependencies.

## Data Availability

The datasets used in the study are not publicly available due to trade secrets of company. In case of requests for access to data, please contact the corresponding author.
